# Fibroblasts as a ruler of the immune microenvironment: measurement and modulation in tissue homeostasis and disease

**DOI:** 10.3389/fimmu.2025.1752621

**Published:** 2026-01-16

**Authors:** Yanling Zhang, Xinyi Fang, Lian Yan, Lin Wang

**Affiliations:** 1 Department of Gastrointestinal Surgery, Sichuan Academy of Medical Sciences & Sichuan Provincial People’s Hospital, University of Electronic Science and Technology of China, Chengdu, China; 2 University of Electronic Science and Technology of China, Chengdu, China; 3 Department of Laboratory Medicine, Ya’an People’s Hospital, Ya’an, China

**Keywords:** cancer-associated fibroblasts, fibroblasts, fibrosis, heterogeneity, immune microenvironment, immunotherapy, single-cell omics, spatial organization

## Abstract

Fibroblasts, once considered merely passive structural components of tissues, are now recognized as dynamic regulators of the immune microenvironment. Recent advances in single-cell and spatial multi-omics have revealed their profound heterogeneity, spatial organization, and functional plasticity, positioning them as a ‘ruler’ that measures, defines, and shapes local immune responses. In both homeostasis and disease contexts—such as cancer, autoimmune disorders, and fibrosis—distinct fibroblast subpopulations exhibit specialized roles: some drive immunosuppression via PD-L1 expression, TGF-β secretion, or metabolic reprogramming; others promote inflammation or fibrosis through cytokine and chemokine secretion; while a subset supports immune resolution and tissue repair. Spatially, fibroblasts organize immune territories by forming physical and chemical barriers, orchestrating tertiary lymphoid structures, and partitioning inflammatory zones. Their bidirectional crosstalk with immune cells—including T cells, macrophages, and B cells—further fine-tunes immune activation or suppression. The dysregulation of fibroblast subsets is a hallmark of disease progression and therapy resistance. Emerging therapeutic strategies aim to ‘recalibrate’ this dysfunctional ruler through targeted depletion, phenotypic reprogramming, or disruption of pathogenic signaling. Integrating fibroblast-centric metrics into clinical practice may enable precise assessment of the immune microenvironment and personalized interventions, heralding a new era in immunotherapy and fibrotic disease management.

## Introduction

In various pathological processes such as infection, autoimmunity, fibrosis, and cancer, the disruption of tissue microenvironment homeostasis is central to disease onset and progression. This microenvironment constitutes a complex ecosystem formed by parenchymal cells, immune cells, and mesenchymal cells (1). For a long time, immune cells were regarded as the primary protagonists in this context, while fibroblasts were merely categorized as passive supporting actors maintaining tissue structure (2). However, over the past five years, breakthroughs in single-cell multi-omics technologies, such as single-cell RNA sequencing and spatial transcriptomics, have fundamentally overturned this traditional understanding. Increasing evidence indicates that fibroblasts are key immune regulators characterized by high heterogeneity and dynamic plasticity. They can not only build tissue structure but also be used to measure, define, and shape local immune responses (3).

Across different tissues (such as the intestine, lung, skin, and liver) and even within distinct microdomains of the same tissue (e.g., the papillary and reticular layers of skin), fibroblasts exhibit unique transcriptional profiles and functional roles. These differences are pre-programmed during development by factors such as HOX genes. Under pathological conditions, this heterogeneity is further amplified and reconfigured. For instance, in rheumatoid arthritis (RA) and inflammatory bowel disease (IBD), distinct fibroblast subpopulations drive either the persistence or resolution of inflammation (3); In dermatological conditions like psoriasis and atopic dermatitis, specific fibroblast subpopulations precisely recruit Th17, Th1, or Th2 cells by secreting distinct chemokines (e.g., CCL19, CXCL13), thereby determining the inflammatory phenotype (4); In tumors, cancer-associated fibroblasts (CAFs) further differentiate into distinct subpopulations, such as pro-fibrotic myofibroblastic-like CAFs (myCAFs) and immunosuppressive CAFs (iCAFs), collectively constructing an inhibitory tumor immune microenvironment (5). This functional diversity positions fibroblast subpopulations and their molecular signatures as novel biomarkers for predicting disease prognosis and immunotherapy response. For instance, in lung cancer, prognostic models built on immune and CAF-related genes have demonstrated exceptional predictive performance (6).

Techniques such as spatial transcriptomics and multicolor fluorescence have demonstrated that fibroblasts are not uniformly distributed; rather, they delineate functionally distinct ‘territories’ within tissues by creating physical barriers, such as dense extracellular matrix, and chemical gradients, such as chemokines (7). In the context of arthritis, pro-inflammatory MMP3+/IL6+ fibroblasts aggregate with inflammatory immune cells, whereas pro-regressive CD200+/DKK3+ fibroblasts establish a remission-promoting microenvironment alongside type 2 innate lymphoid cells (ILC2s) (8). At tumor margins, specific subpopulations of cancer-associated fibroblasts (CAFs), such as CTHRC1-expressing fibroblasts, create a pro-fibrotic niche in conjunction with macrophages, forming a physical and immunological barrier that excludes cytotoxic T cells. More critically, fibroblasts act as primary organizers of tertiary lymphoid structures (TLSs). By secreting key chemokines like CXCL13, they recruit lymphocytes and facilitate their compartmentalization, thereby establishing a local immune activation and regulatory hub within chronic inflammation and tumor microenvironments. The maturity of these TLSs is closely correlated with patient responses to immune checkpoint inhibitors (9).

Ultimately, fibroblasts adjust the immune response dynamically through a complex intercellular communication network. They engage in bidirectional dialogues with nearly all immune cell types: directly suppressing T cell function and promoting regulatory T cell (Treg) expansion by expressing PD-L1 and secreting factors such as TGF-β and IL-6 (10); secreting factors like CSF1 and GM-CSF to “educate” myeloid cells, driving macrophage polarization towards M2-like pro-tumor or pro-fibrotic phenotypes—a vicious cycle observed in giant cell arteritis (GCA); and stabilizing the ILC2 phenotype via the CD200-CD200R signaling axis, actively promoting inflammation resolution (8). In breast cancer, CAF-associated long non-coding RNAs (e.g., LINC00844) exhibit expression patterns significantly correlated with reduced CD8+ T cell infiltration and increased M2 macrophages, profoundly influencing drug sensitivity (10).

This review systematically examines the potential and mechanisms of fibroblasts adjust the immune microenvironment. We will explore how their heterogeneity measures immune status, how their spatial distribution defines the region of immune cell activity, and how their dynamic interactions precisely regulate the intensity and direction of immune responses. Finally, we will outline novel therapeutic strategies targeting fibroblasts to regulate dysregulated immune microenvironments, aiming to provide fresh perspectives and directions for future research.

## The scale’s graduations: functional heterogeneity of fibroblasts

Traditionally, fibroblasts were perceived as a homogeneous and functionally uniform cell population. However, revolutionary advances in single-cell multi-omics technologies—such as single-cell RNA sequencing and spatial transcriptomics—have completely transformed this understanding. These technologies function like high-powered magnifying glasses, revealing fibroblasts as a complex ecosystem composed of functionally distinct and molecularly diverse subpopulations. It is precisely these specific subpopulations and their changing proportions that indicate whether the microenvironment is in an immunologically activated, immunosuppressed, or inflammation-resolving state, thereby influencing disease progression and therapeutic response (11, 12). This cognitive leap in understanding fibroblast heterogeneity began with the widespread adoption of single-cell analysis technologies. Early batch sequencing studies could only capture average signals across populations, obscuring significant intercellular variation. In contrast, scRNA-seq unbiasedly resolves the entire transcriptome at single-cell resolution, identifying discrete subpopulations with unique gene expression profiles within seemingly homogeneous fibroblast populations. For instance, in pancreatic ductal adenocarcinoma, studies first identified at least three major cancer-associated fibroblast (CAF) subpopulations: myofibroblast-like CAFs, inflammatory CAFs, and antigen-presenting CAFs. These subpopulations exhibit high expression of α-SMA, IL-6, and MHC class II molecules, respectively, playing distinctly different and even opposing roles in tumor progression (13). Similarly, studies utilizing single-cell RNA sequencing (scRNA-seq) have identified multiple fibroblast subpopulations, including subchondrocyte-like fibroblasts that promote bone erosion and PRIME cells that drive inflammation (12, 14). Immunosuppressive Subpopulations: This subset is the most extensively studied and prevalent in cancer and chronic inflammation. They effectively suppress effector immune cells, such as T cells, through multiple mechanisms. Key characteristics include high expression of immune checkpoint ligands (e.g., PD-L1) and the secretion of abundant inhibitory cytokines, such as transforming growth factor-β and interleukin-10 (15). For instance, specific cancer-associated fibroblast (CAF) subpopulations found in various cancer types can generate high local concentrations of adenosine by secreting prostaglandin E2 or expressing exonucleases like CD73, which directly inhibit CD8+ T cell activity and proliferation (16, 17).

Tissue Structure-Remodeling Subpopulations The primary function of this subpopulation is the extensive synthesis and remodeling of the extracellular matrix, which influences immune cell infiltration by establishing physical barriers. A prominent example of this is myofibroblast-like cancer-associated fibroblasts (CAFs), which exhibit high expression levels of α-smooth muscle actin and type I collagen, thereby creating a dense fibrotic stroma. This physical barrier not only hinders the delivery of chemotherapy drugs but, more critically, restricts the infiltration of cytotoxic T cells into the tumor parenchyma, resulting in a spatially defined “immune-privileged” zone. The significance of this “scale” directly quantifies the microenvironment’s physical permeability and the extent of immune exclusion (18, 19).

Immune Homeostasis-Maintaining Subpopulations It is noteworthy that not all fibroblast subpopulations contribute to pathological processes. During tissue homeostasis and the resolution of inflammation, certain subpopulations demonstrate positive immunoregulatory functions. For instance, the CD200+ fibroblast subpopulation identified in arthritis models transmits inhibitory signals by interacting with CD200R on immune cells, thereby promoting the resolution of inflammation. In the gut, specific collagen-producing fibroblasts support the function of regulatory T cells and type 2 innate lymphoid cells by expressing IL-33, thus maintaining mucosal barrier immune homeostasis. The strength of the immune homeostasis-maintaining subpopulations serves as a crucial indicator for evaluating tissue repair capacity and the timely resolution of inflammation (8, 20).

Driven by microenvironmental signals such as cytokines (e.g., TGF-β, IL-1), mechanical stress, and metabolites, fibroblasts can transition between different subpopulations (21). For instance, in pancreatic cancer, TGF-β signaling drives fibroblast differentiation toward myofibroblast-associated CAFs (myCAFs), while IL-1 promotes their transition to inflammatory CAFs (iCAFs). This plasticity implies that fibroblasts which can be used for measure the immune microenvironment can be adjusted by disease signals and calibrated by therapeutic interventions (22). Therefore, understanding and targeting these specific “markers”—such as developing small-molecule drugs to deplete immunosuppressive CAFs, using anti-fibrotic agents to weaken the function of the barrier subpopulation, or employing cytokine-neutralizing antibodies to reprogram harmful subpopulations toward beneficial phenotypes—emerges as a highly promising therapeutic strategy (23).

**Figure 1:**
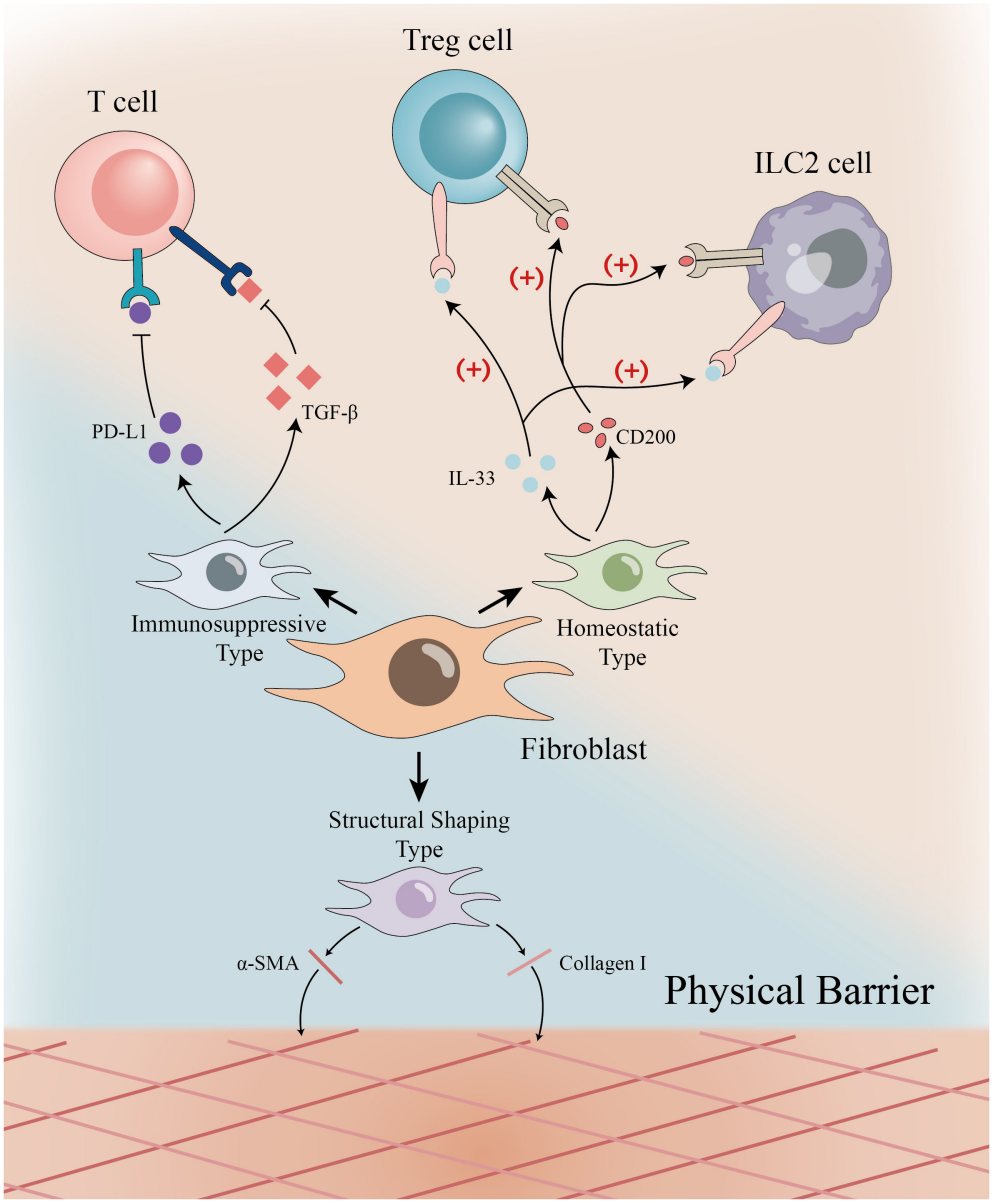
Functional heterogeneity of fibroblasts—the “scale” of the immune microenvironment fibroblast subpopulations in different tissues exhibit distinct roles in immune regulation: Immunosuppressive (PD-L1^+^, TGF-β^+^) suppress T cells; Structural-plasticity (α-SMA^+^, Collagen I^+^) form physical barriers; Homeostatic (CD200^+^, IL-33^+^) support Treg/ILC2.

In summary, Each distinct, molecularly defined subpopulation corresponds to a precise mark on this ruler, quantitatively or qualitatively reflecting the local immune state. Deep decoding of these “markings” not only enriches our understanding of disease mechanisms but also provides a novel roadmap and targets for developing precise interventions within the immune microenvironment (**Figure 1**).

## The compass pointing: spatial distribution of fibroblasts and immune compartmentalization

By creating specific spatial niches, fibroblasts physically manifest the heterogeneity of the immune microenvironment, thereby influencing the location, intensity, and ultimate outcome of immune responses (24, 25).

In solid tumors, one of the most significant spatial functions of cancer-associated fibroblasts (CAFs) is the formation of an immune exclusion barrier at the tumor-normal tissue interface or surrounding tumor nests (26). This function arises not from a singular mechanism but from the synergistic action of both physical and chemical barriers (27).

Physical Barrier: Primarily mediated by myofibroblast-like cancer-associated fibroblasts (myCAFs), which are driven by signals such as TGF-β. These cells excessively produce and cross-link extracellular matrix (ECM) components, such as type I collagen and fibronectin, resulting in a dense and highly organized fibrotic stroma (28). This proliferated matrix acts as an ‘armor’ for tumor cells, significantly increasing tissue stiffness. Consequently, this not only impedes the physical migration of effector immune cells, such as cytotoxic T lymphocytes (CTLs) and natural killer (NK) cells, but may also suppress T cell function through inhibitory signals mediated by integrin signaling (29).

Chemical Barriers Specific subpopulations of cancer-associated fibroblasts (CAFs) create a spatial “immune desert” or a “misdirected” chemotactic environment by secreting distinct chemokines. For instance, certain CAF subpopulations exhibit high expression levels of CXCL12, whose receptor, CXCR4, is commonly found on T cells and myeloid cells. However, this CXCL12 gradient does not effectively recruit T cells to the tumor core; rather, it may sequester or repel them to the stromal zone at the tumor periphery, thereby preventing effective engagement with cancer cells. Meanwhile, this renders a substantial number of T cells in a state of ‘ineffective infiltration’. Moreover, the high levels of CXCL12 signaling within the tumor microenvironment may further directly or indirectly promote lymphatic vessels also transport tumor-specific effector T cells out of tumors. This systematically and persistently depletes the number of T cells within the tumor, thereby preventing the establishment and maintenance of functional immune foci (30).

Furthermore, CAFs locally overexpress metabolic immunosuppressive molecules such as prostaglandin E2 (PGE2) and adenosine, creating a chemical “exemption zone” where any T cells that manage to infiltrate rapidly lose functionality (31, 32). Recent studies employing multicolor immunofluorescence and spatial transcriptomics have visually elucidated the structure of this barrier. Apart from that, Recent studies have revealed that CAFs not only form a barrier but also actively regulate tumor cell biological behavior. For example, In colorectal cancer (CRC), CTHRC1 overexpresses WNT5A protein, which promotes epithelial-mesenchymal transition (EMT) and enhances tumor cell invasiveness by upregulating MSLN expression in adjacent malignant epithelial cells. This signaling axis—CTHRC1+ CAF-WNT5A-MSLN—plays a crucial role in the progression and metastasis of CRC) (33).

Tissue Immune Specialized Zones: Formation and Maintenance of Tertiary Lymphoid Structures (TLS). In contrast to the role of fibroblasts in tumor barrier construction, these cells serve a diametrically opposed function in chronic infections, autoimmune diseases, and certain tumors that respond to immunotherapy, acting as a major component of tertiary lymphoid structures (TLS). TLS are highly organized aggregates of lymphocytes that develop ectopically at sites of chronic inflammation, functioning as the origin of local adaptive immune responses (3). Lymphocytes recruited to the tumor activate lymphotoxin-β receptor (LTβR), inducing TLS tissue fibroblasts to express adhesion molecules such as VCAM-1 and ICAM-1 and produce chemokine gradients (such as CXCL13, CCL21). This facilitates the directed recruitment, precise localization, and compartmentalization of lymphocytes (T cells, B cells), leading to the formation of T cell zones and B cell follicles. Simultaneously, TLS fibroblasts drive tumor vascular normalization. By secreting vascular stabilizing factors and providing physical support, they assist in constructing and maintaining this functionally competent vascular network, thereby ensuring the long-term survival and sustained activation of immune cells within the TLS (34). The presence of TLS is typically associated with improved prognosis and responses to immune therapy. Thus, the spatial localization of fibroblast subpopulations capable of organizing and maintaining TLS directly indicates immunologically activated “hotspot” regions with the potential to generate robust antitumor or anti-pathogen immune responses. Their abundance and distribution serve as crucial spatial indicators for assessing the immune competence of the microenvironment (35, 36).

Mapping Inflammatory Landscapes: Micro-Segregation in Chronic Inflammation and Autoimmune Diseases. In chronic inflammatory diseases such as rheumatoid arthritis (RA) and inflammatory bowel disease (IBD), the role of fibroblasts in spatial partitioning is crucial. They no longer act as uniform participants in inflammation; rather, they differentiate into functionally specialized subpopulations within distinct anatomical microenvironments, driving markedly different pathological processes. In RA, synovial fibroblasts maintain their activated and invasive state via the Notch signaling pathway, functioning as key effector cells and regulators of the inflammatory microenvironment. Their characteristic ITGA5+ subpopulation directly contributes to articular cartilage erosion and perpetuates chronic inflammation by remodeling the extracellular matrix and establishing a pro-inflammatory microenvironment. In contrast, intestinal fibroblasts in IBD primarily undertake tissue repair functions. Their pathological abnormalities mainly manifest as excessive extracellular matrix deposition and fibrosis mediated by signaling pathways such as TGF-β, leading to intestinal strictures rather than direct active tissue destruction (37, 38).

Invasive Frontiers vs. Inflammatory Zones: In the synovium of rheumatoid arthritis, spatial analysis indicates that LINCO1846+ subchondral fibroblasts preferentially localize at the cartilage-bone junction. Here, they express high levels of RANKL and MMPs, interact with plasma cells, and thereby strongly promote osteoclastogenesis and periarticular bone erosion (39). In contrast, PRIME cells and HLA-DRAhi inflammatory fibroblasts are enriched in the synovial lining zone, directly sustaining chronic inflammation by producing abundant cytokines and chemokines (such as IL-6 and CCL2) that promote macrophage and lymphocyte infiltration. They also participate in abnormal tissue repair and fibrosis following tissue destruction by regulating immune cell localization and activation (40). This spatially compartmentalization allows different fibroblast subpopulations to function simultaneously at the bone destruction and the inflammation maintenance.

Mucosal Barrier and Homeostasis: In the gut, specific collagen-type fibroblasts located at the base of crypts express IL-33 (41), providing a supportive microenvironment for type 2 innate lymphoid cells (ILC2s) and regulatory T cells (Tregs) through the ST2 receptor signaling pathway. This expression is crucial for maintaining mucosal barrier homeostasis and facilitating repair processes. The spatial positioning of these fibroblasts determines their functional specificity (42).

**Figure 2:**
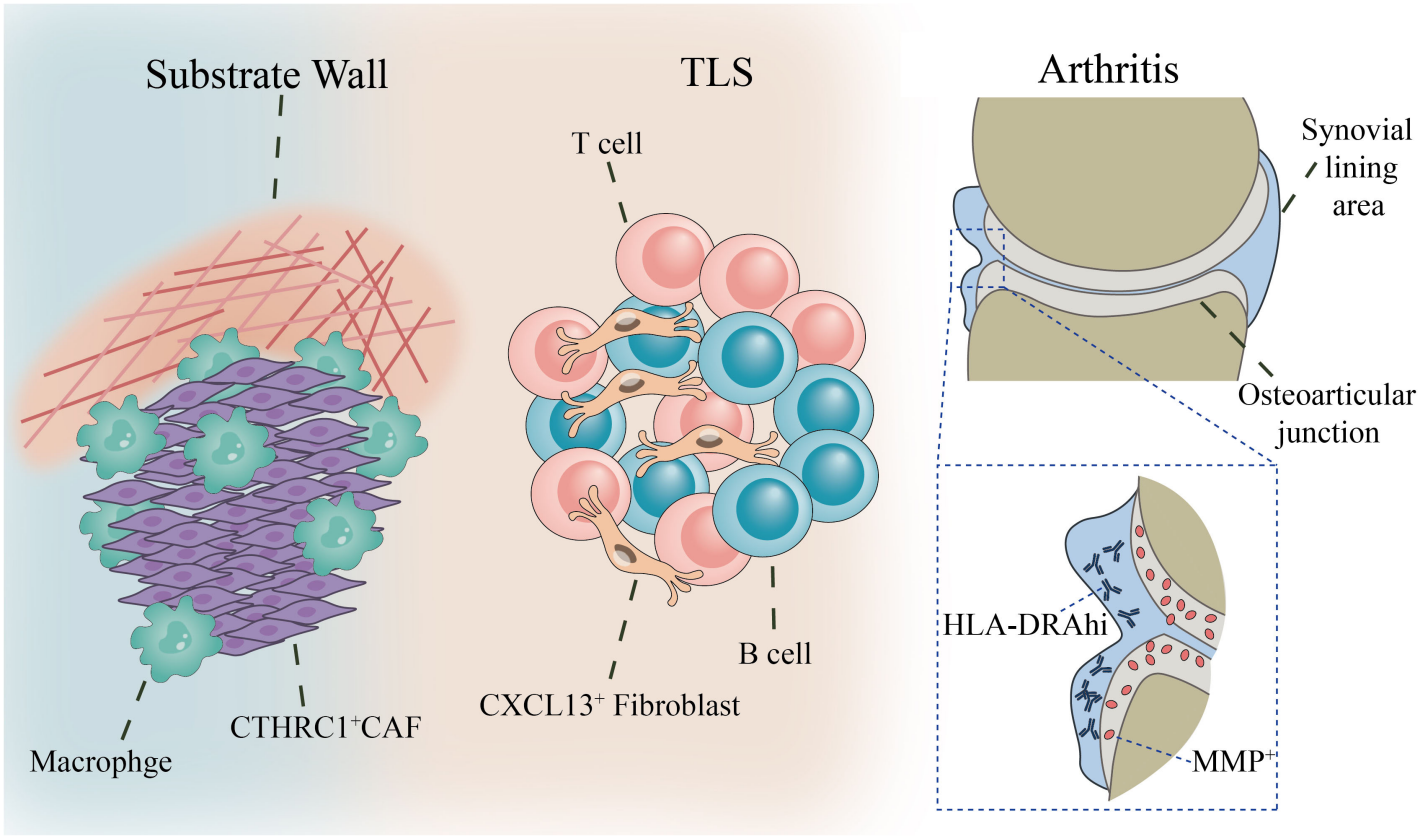
Spatial distribution of fibroblasts and immune compartmentalization. Spatial localization of fibroblast subpopulations in distinct regions and their influence on immune cell (T cells, macrophages, B cells) distribution. In tumors, CAFs form a “matrix wall” at the invasion front (CTHRC1^+^ CAFs + macrophages); in chronic inflammation, TLS structures (CXCL13^+^ fibroblasts + B/T cell zones); In arthritis, spatial separation between the synovial lining zone (HLA-DRAhi) and the bone interface zone (MMP^+^).

In summary, fibroblasts are not merely uniform fillers scattered throughout tissues. Through their specific spatial distribution, they actively partition tissues into functionally distinct immune zones (11)—whether forming immunosuppressive barrier (43), assembling supportive lymphoid aggregates (TLS) (44), or partitioning functionally specialized inflammatory niches (25). It is precisely this capability for precise spatial orientation that enables fibroblasts to became the core force that defines, shapes, and ultimately controls the local immune microenvironment. Understanding this spatial logic provides fundamental guidance for developing next-generation therapies capable of breaching physical barriers or precisely regulating local immune responses (45) (**Figure 2**).

## Turning the dial: dynamic interactions between fibroblasts and immune cells

The dynamic regulatory process is a highly complex, bidirectional intercellular signaling network established between fibroblasts and various immune cells. These interactions function like countless invisible hands that collectively adjust the balance of the gauge, ultimately determining whether the microenvironment favors immune attack, immune tolerance, or immune suppression. A thorough examination of these dynamic interactions is crucial for understanding the biological functions of fibroblasts and for developing effective intervention strategies.

Direct Suppression and Indirect Exhaustion: Precision Regulation of T Lymphocytes. T lymphocytes, particularly cytotoxic CD8^+^ T cells, play a central role as effectors in anti-tumor and anti-viral immunity. Fibroblasts utilize multiple mechanisms to ‘precisely suppress’ these cells, directly shifting their functional response towards inhibition (46). Immune Checkpoint Ligand Expression: Numerous cancer-associated fibroblast (CAF) subpopulations overexpress PD-L1. When PD-L1 binds to the PD-1 receptor on the surfaces of T cells, it transmits inhibitory signals that impair T cell proliferation, reduce cytokine secretion, and diminish cytolytic function, thereby inducing T cell ‘exhaustion.’ This mechanism allows CAFs to directly disable infiltrating T cells, similar to the action of tumor cells (27). Metabolic Reprogramming and Nutrient Deprivation: Fibroblasts indirectly suppress T cells by modulating the local metabolic environment. For instance, CAF subpopulations that highly express CD73 and CD39 catalyze the conversion of extracellular ATP/ADP into adenosine, a potent immunosuppressive agent (47). Adenosine acts on T cells via its A2A receptor, significantly suppressing their activation and function. Additionally, CAFs compete with immune cells for essential amino acids, such as tryptophan and arginine, leading to T cell dysfunction due to ‘nutritional deprivation’ (48). Shaping an Inhibitory Cytokine Environment: Fibroblasts are a primary cellular source of TGF-β, a multifunctional cytokine that not only directly suppresses the effector functions of CD8^+^ T cells and Th1 cells but also powerfully induces the differentiation of naive T cells into regulatory T cells (Tregs). The expansion of Tregs further enhances the local immunosuppressive environment through both cell contact-dependent and independent mechanisms (49).

Educating Myeloid Cells: Dominant Influence on Macrophage Polarization Macrophages are highly plastic cells within the microenvironment, and their functional state—whether pro-inflammatory (M1-like) or anti-inflammatory (M2-like)—profoundly impacts disease progression. Fibroblasts serve as one of the primary educators of macrophage phenotype and function (50). Driving M2-like Polarization Cancer-associated fibroblasts (CAFs) continuously secrete factors such as CSF1, IL-6, and CCL2, which not only recruit monocytes to the lesion site but also drive their polarization toward an M2-like phenotype, characterized by tissue repair and immunosuppressive functions. In turn, M2-like macrophages secrete factors like TGF-β and IL-10 to further activate and sustain the CAF phenotype, forming a positive feedback loop between CAFs and macrophages that collectively constructs a robust immunosuppressive niche (51).

Mediating Vascular Inflammation and Injury: In vasculitic diseases such as giant cell arteritis, a vicious interaction mediated by GM-CSF exists between fibroblasts and macrophages within the vascular wall. GM-CSF produced by fibroblasts drives macrophages to generate pro-inflammatory factors like TNF-α, which subsequently activate fibroblasts, leading to sustained inflammatory amplification and vascular damage (52).

Supporting Humoral Immunity Hubs: Symbiosis with B Cells and Tertiary Lymphoid Structures. In chronic inflammation and certain tumors, fibroblast-B cell interactions are crucial for the formation and maintenance of tertiary lymphoid structures (TLS) (53). Fibroblasts expressing CXCL13 play a central role in recruiting CXCR5^+^ B cells into TLS (54). They not only secrete chemokines but also provide survival and retention signals for B cells by expressing adhesion molecules such as VCAM-1 and ICAM-1. In structurally mature TLS, fibroblasts—particularly those exhibiting follicular dendritic cell (FDC)-like characteristics—directly support B cell activation and proliferation by expressing factors such as BAFF and APRIL. This promotes high-efficiency antibody production, shifting the immunological microenvironment toward an “activated” state (55).

Novel Communication Modalities: Extracellular Vesicles and Long-Range Signaling Beyond soluble factors, fibroblasts engage in long-range intercellular communication by releasing extracellular vesicles (EVs), including exosomes and micro-vesicles. These vesicles carry parent-cell-derived proteins, lipids, mRNA, and non-coding RNAs (e.g., miRNA, lncRNA), which can be internalized by immune cells to modulate their functional states. For instance, CAF-derived exosomes enriched with immunosuppressive miRNAs can remotely suppress T cell activation (56). This “encapsulated” signaling significantly expands the spatiotemporal scope of fibroblast-mediated immune regulation.

Fibroblasts engage in spatiotemporally specific dynamic interactions with the immune system across multiple tissues. Those distributed at tissue boundaries, such as the brain, are activated by TGFβ signaling upon injury to become myofibroblasts. These myofibroblasts then collaborate with macrophages and other cells to regulate repair and scar formation. Following brain injury, they initially limit inflammation and later shape the immune microenvironment by secreting chemokines to recruit T cells. This interaction axis offers novel therapeutic strategies for disease management (49, 57).

**Figure 3:**
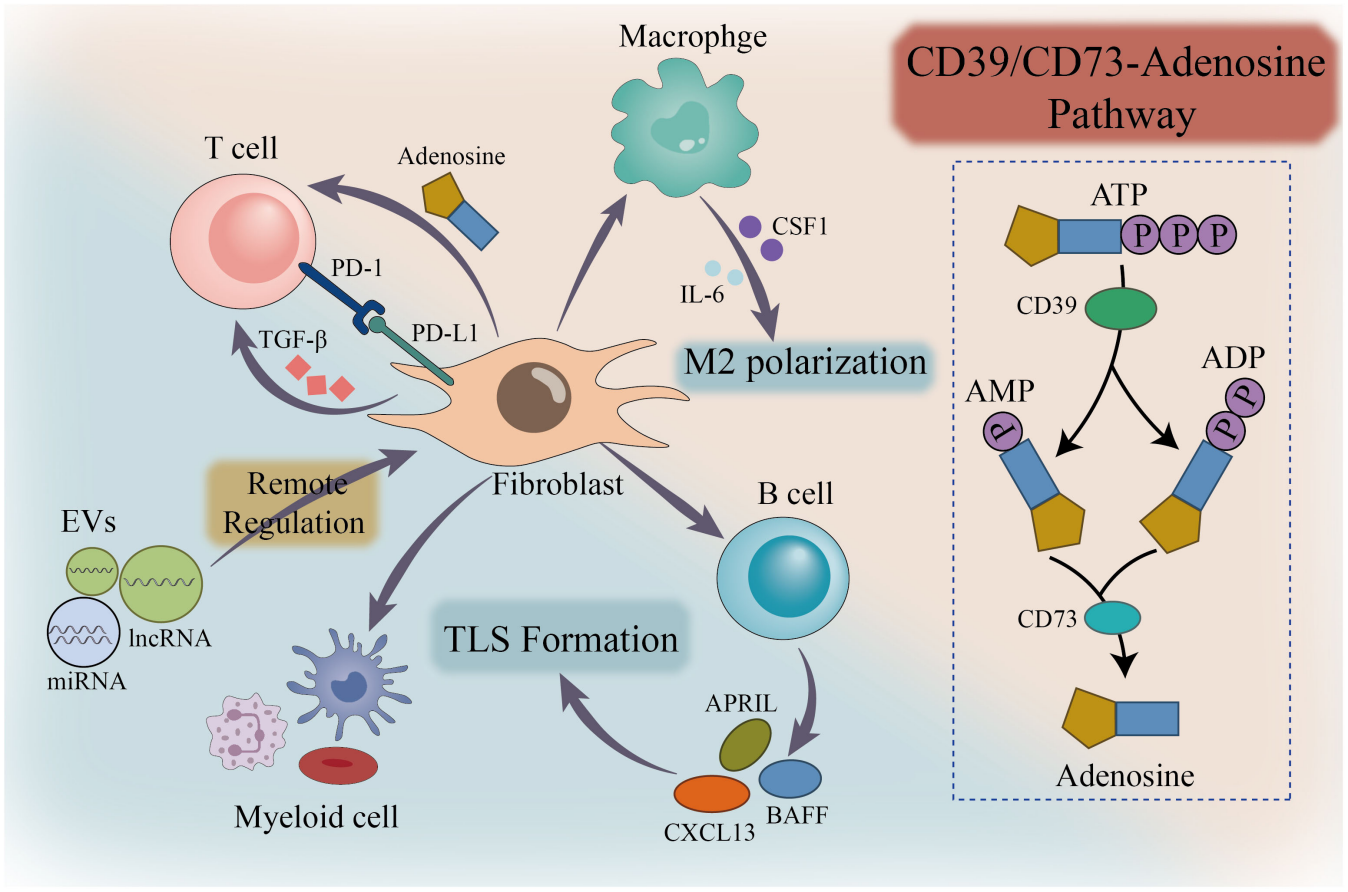
Dynamic interaction network between fibroblasts and immune cells. Interactions between fibroblasts and major immune cells (T cells, macrophages, B cells, myeloid cells).They induce T cell differentiation via PD-L1/PD-1, adenosine, and TGF-β; promote M2 polarization of macrophages through CSF1 and IL-6; influence B cells via CXCL13 and BAFF/APRIL to facilitate TLS formation; and enable remote regulation by carrying miRNAs/lncRNAs within extracellular vesicles (EVs).

Fibroblasts engage in continuous dynamic dialogues with key immune players, including T cells, macrophages, and B cells, through a multifaceted mechanism that encompasses direct contact, soluble factors, metabolic regulation, and extracellular vesicles. Each transmission and reception of signals acts as a “tweak” on the scale of the immune microenvironment, subtly adjusting the balance of immune responses. Understanding the molecular details of these interactions not only reveals the underlying mechanisms of disease development but also provides numerous potential intervention targets. By precisely disrupting harmful dialogues or enhancing beneficial exchanges through drugs, antibodies, or cell therapies, we can recalibrate dysregulated immune microenvironments towards health or a state capable of effectively combating disease (**Figure 3**).

## The imbalance of the ruler: a critical role in disease onset and progression

Under normal conditions, they maintain the equilibrium of immune responses and tissue homeostasis. However, under sustained pathological stressors—such as chronic inflammation, genetic mutations, or tissue injury—these “rulers” undergo profound dysfunction, resulting in imbalances among specific functional subpopulations, spatial mislocalization, and disrupted communication networks. This “ruler imbalance” transcends being a mere consequence of disease; it becomes a core driver that propels disease onset, progression, and ultimately determines clinical outcomes.

Cancer: Within the tumor microenvironment, fibroblast imbalance is most characteristically manifested as the expansion of immunosuppressive and pro-fibrotic subpopulations, coupled with the depletion or silencing of immune-supportive subpopulations. This imbalance firmly drives the microenvironment toward a immunosuppressive state, specifically reflected in: Across multiple cancer types (e.g., pancreatic, breast, colorectal), TGF-β-secreting myCAFs and IL-6-secreting iCAFs frequently undergo excessive proliferation and activation (58). Through these mechanisms, they collaboratively construct a microenvironment that is both physically impermeable and chemically toxic. For instance, CAFs expressing FAP correlate strongly with reduced CD8+ T cell infiltration and poor prognosis; conversely, CAFs secreting CXCL12 create a chemical barrier that excludes T cells from tumor nests (59). This abnormal accentuation of the specific immunosuppression represents a critical step in tumor immune evasion.

Pathological Spatial Remodeling: The imbalanced spatial distribution of CAFs further entrenches immunosuppression. Instead of supporting antitumor immunity around tertiary lymphoid structures (TLS), they form dense “matrix walls” at the tumor invasion front. This misdirected spatial orientation physically isolates tumor cells from immune attacks, directly contributing to immunotherapy failure (60).

As Biomarkers for Prognosis and Treatment Response: The dysregulated state of fibroblasts has significant clinical implications. Studies demonstrate that prognostic models based on cancer-associated fibroblast (CAF)-associated gene or long non-coding RNA (lncRNA) signatures effectively predict survival in patients with colorectal adenocarcinoma, breast cancer, and other malignancies. A highly immunosuppressive and fibrotic CAF profile typically indicates a poor response to immune checkpoint inhibitors, such as PD-1 antibodies. Merely ‘releasing the T-cell brakes’ is insufficient to reverse the overall suppressive landscape.

Autoimmune Diseases and Chronic Inflammation: In conditions such as rheumatoid arthritis, inflammatory bowel disease, and psoriasis, an imbalance in fibroblasts is characterized by an abnormal fixation to a pro-inflammatory phenotype and a loss of anti-inflammatory or anti-exacerbation functions (61).

Recent studies have identified a unique subpopulation of fibroblasts at inflammatory sites that maintain long-term pathological memory, termed “pathologically imprinted fibroblasts” (62). For instance, in rheumatoid arthritis, specific fibroblast subpopulations undergo epigenetic reprogramming following inflammatory stimulation. Even after inflammation temporarily subsides, these cells retain this reprogrammed state, enabling them to produce pro-inflammatory factors more rapidly and robustly upon subsequent stimulation (63). This “molecular memory” causes the regulatory balance to persistently shift toward inflammation after the acute phase concludes, contributing to the chronicity and recurrence of the disease.

Erroneous Segmentation of Inflammatory Micro-zones: As previously described, in the rheumatoid arthritis synovium, pro-inflammatory HLA-DRAhi fibroblasts occupy the lining layer, while MMP-expressing erosive fibroblasts localize at the bone interface (64). This spatial imbalance facilitates the precise execution and continuous amplification of inflammatory responses and tissue destruction across two distinct anatomical zones, ultimately driving irreversible joint damage (65).

Disruption of Inflammatory Resolution Mechanisms: Under steady-state conditions, pro-resolution fibroblast subpopulations, such as CD200+ cells, emit “stop” signals at appropriate junctures. However, during chronic inflammation, these subpopulations may diminish in number or become functionally suppressed, hindering timely resolution and leading to a failure in the self-calibration function of the inflammatory scale (8).

Fibrotic Diseases: In idiopathic pulmonary fibrosis, liver fibrosis, and systemic sclerosis, the fundamental imbalance in fibroblasts is characterized by their persistent and uncontrolled activation, along with excessive matrix deposition (66, 67).

Apoptosis Resistance and Persistent Activation: In normal tissue repair, activated myofibroblasts typically undergo apoptosis and are cleared after fulfilling their role. However, in fibrotic diseases, these cells develop resistance to apoptosis and remain in an activated state, driven by signals such as PDGF, CTGF, and self-produced TGF-β (68).

Synergistic Polarization of the Immune Microenvironment: Pathogenic fibroblasts and M2 macrophages engage in a vicious cycle in pulmonary fibrosis. Factors secreted by fibroblasts, such as CCL18, recruit and polarize macrophages, while M2 macrophages further activate fibroblasts by secreting factors like TGF-β. Together, they drive the microenvironment towards “over-repair”—the precipice of fibrosis—ultimately resulting in structural destruction and functional failure of the organ (69, 70).

**Figure 4:**
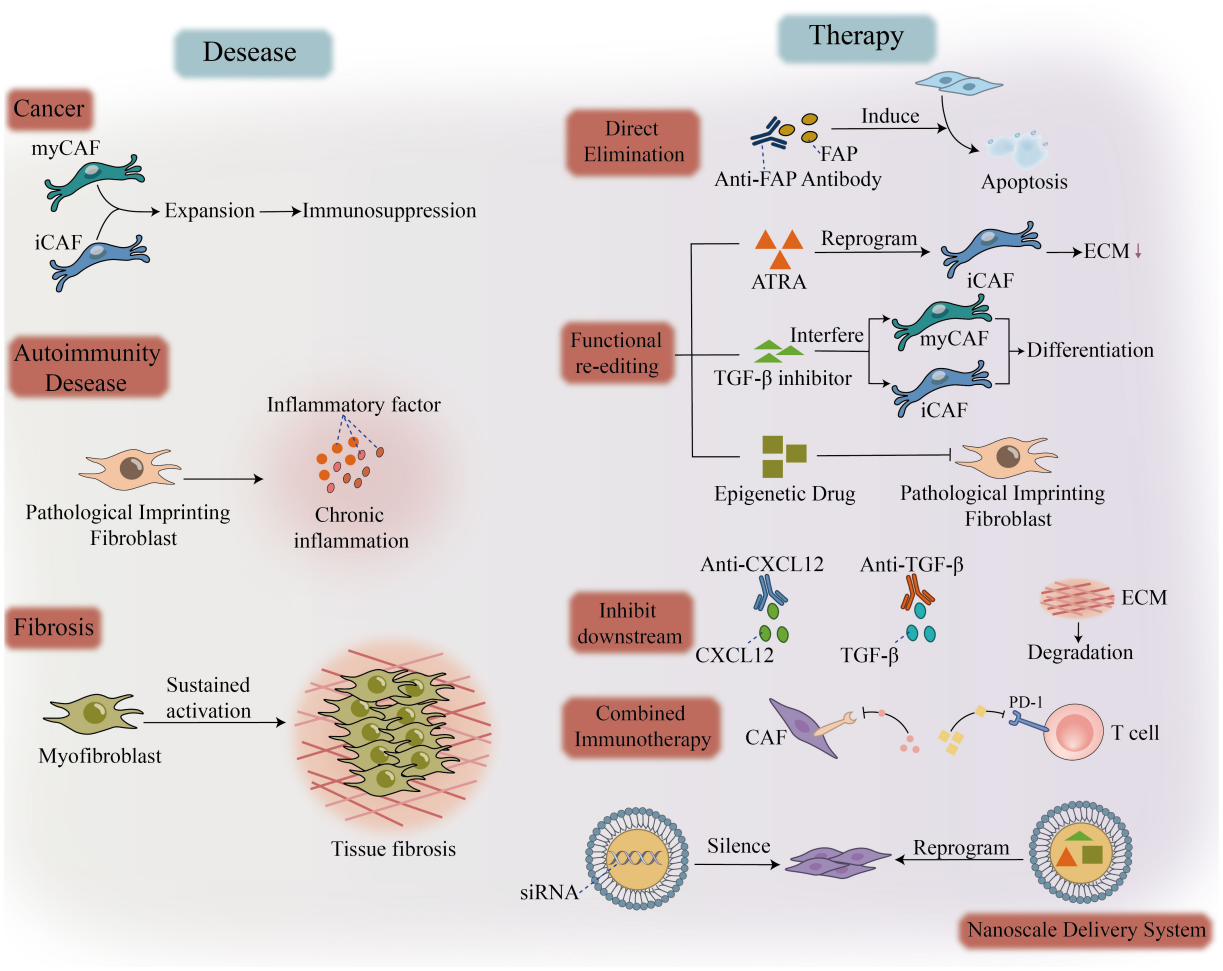
Dysregulation of fibroblasts in disease and therapeutic calibration strategies. Disease-associated fibroblast dysregulation leads to immune suppression via myCAF/iCAF amplification in the tumor microenvironment; pathologically imprinted fibroblasts drive persistent chronic inflammation in autoimmune diseases; tissue fibrosis promotes sustained myofibroblast activation resulting in tissue fibrosis. Therapeutic approaches targeting fibroblasts can restore these imbalanced immune microenvironments, including: - Direct elimination: anti-FAP antibodies, apoptosis induction - Functional reprogramming: ATRA, TGF-β inhibitors, epigenetics drugs - Downstream blockade: anti-CXCL12, anti-TGF-β, ECM degradation - Combined immunotherapy: CAF targeting + anti-PD-1.

In summary, the imbalance of fibroblasts serves as a critical benchmark for the immune microenvironment across various major diseases. Whether the shift leads to immunosuppression, chronic inflammation, or fibrosis, the essence lies in the disruption of the steady state of fibroblast functional subpopulations across three dimensions: quantity, spatial distribution, and temporal dynamics. Understanding the specific patterns of this imbalance—identifying where the “calibration” is flawed, the origins of the problem, and the reasons for its persistence—not only elucidates the root causes of disease persistence but also highlights precise therapeutic targets (**Figure 4**).

## A novel therapeutic strategy targeting fibroblasts

When the calibration scale of the immune microenvironment indicates a pathological state due to dysregulated fibroblast function, the primary therapeutic approach is to restore it to homeostasis. Traditional therapies, such as broad-spectrum anti-inflammatory drugs or cytotoxic chemotherapy, may temporarily alter superficial immune parameters but fail to correct the dysfunctional cellular and molecular architecture of the microenvironment, and may even exacerbate damage due to their non-specific nature. With increasing insights into fibroblast heterogeneity and functional mechanisms, therapeutic strategies are experiencing a paradigm shift: transitioning from indiscriminate attacks to precision interventions. These interventions aim to directly adjust dysregulated fibroblast subpopulations or functions, thereby reshaping the immune microenvironment to promote disease resolution. These novel calibration strategies represent the forefront of treatment in oncology, rheumatology, and fibrotic diseases.

Direct Clearance: Depleting Pathogenic Subpopulations. The most direct calibration strategy involves the removal of problematic “calibration points” by selectively depleting pathogenic fibroblast subpopulations that are excessively expanded or activated in disease (71). Antibody-Mediated Cytotoxicity Monoclonal antibodies targeting membrane proteins that are highly expressed on specific CAF subpopulations eliminate these cells through antibody-dependent cellular cytotoxicity (ADCC) or complement-dependent cytotoxicity (CDC). Antibodies against fibroblast activation protein (FAP), for instance, have been extensively studied. Despite facing challenges in early clinical trials due to off-target toxicity and target heterogeneity, next-generation FAP-targeting bispecific antibodies or antibody-drug conjugates (ADCs) are demonstrating improved precision. FAP-targeted radionuclide therapy has achieved measurable responses in patients with refractory end-stage cancer, and adverse events are manageable, MP0317 is a DARPin^®^ candidate targeting fibroblast activation protein (FAP) and CD40. This study will examine blood levels of MP0317 across several escalating dose levels and determine the recommended dose for further development. The recommended dose will be tested in the second part of the study to confirm safety and further evaluate preliminary biological and antitumor activity. Administration of AMD3100, an inhibitor of chemokine (C-X-C motif) receptor 4 (CXCL12 receptor), also demonstrated the antitumor efficacy of immunotherapeutic antibodies and significantly reduced cancer cells (72, 73). Although these studies have demonstrated significant efficacy in preclinical research and undergone safety evaluations, clinical studies remain lacking to validate these findings.

Small-Molecule Inhibitors and Apoptosis Induction: Developing small-molecule drugs capable of triggering apoptosis in pathogenic fibroblasts is another promising strategy. For example, inhibitors targeting anti-apoptotic proteins such as Bcl-2/xL, as well as compounds capable of inducing myofibroblast apoptosis in fibrotic diseases, are currently being evaluated in preclinical and early clinical studies. The core challenge of this approach lies in distinguishing pathologically activated fibroblasts from normal fibroblasts, which are essential for maintaining tissue homeostasis, thereby avoiding the impairment of tissue repair functions (74, 75).

Functional Reprogramming: Compared to direct elimination, a more sophisticated and potentially safer strategy involves functional reprogramming, which alters the phenotype of cells through pharmacological intervention without inducing cell death. This process transforms tumor-promoting and pro-inflammatory fibroblasts into quiescent states or even phenotypes exhibiting anti-tumor and pro-regressive functions (76, 77).

Targeting Key Signaling Pathways: In pancreatic cancer models, vitamin A derivatives, such as all-trans retinoic acid (ATRA), have been demonstrated to reprogram inflammatory cancer-associated fibroblasts (iCAFs) towards a more quiescent state characterized by reduced extracellular matrix (ECM) production. Similarly, TGF-β receptor inhibitors and IL-1 receptor antagonists can disrupt differentiation signals for myCAFs and iCAFs, respectively, thereby reversing their pathogenic functions (78). Recent evidence indicates that fibroblasts express CD40, which binds to CD40L on activated T cells and macrophages. This interaction further amplifies the production of pro-inflammatory cytokines such as IL-6 and TNF-α, while also influencing the polarization and efficacy of adaptive immunity by regulating the expression of co-stimulatory molecules and cell survival signaling. This mechanism offers novel intervention strategies and potential therapeutic targets for autoimmune diseases, fibrosis, and tumor immunotherapy (79). Epigenetic Regulation: Considering that the ‘pathological imprinting’ of fibroblasts involves epigenetic reprogramming, drugs targeting histone deacetylases (HDACs) or DNA methyltransferases (DNMTs) may offer promise in erasing these pathological memories, thus restoring fibroblasts to a more neutral state (80).

Blocking Downstream Effects: Neutralizing Harmful Outputs. When directly targeting cells proves challenging, an effective alternative is to intercept the harmful “signals” they release—specifically, neutralizing the immunosuppressive or pro-fibrotic factors produced by fibroblasts. Targeting the extracellular matrix: For the dense extracellular matrix (ECM) produced by cancer-associated fibroblasts (CAFs), drugs such as glycosaminoglycan hydrolases (e.g., PEGPH20) degrade hyaluronic acid within the matrix, thereby reducing interstitial pressure and improving the infiltration of drugs and immune cells. Additionally, drugs targeting collagen crosslinking enzymes, such as LOXL2, can disrupt the physical barrier function of the matrix.

Neutralizing immunosuppressive molecules: The development of neutralizing antibodies or receptor traps to block TGF-β, CXCL12, or IL-6 secreted by CAFs is crucial. For instance, combination therapy using CXCL12 neutralizers and PD-1 inhibitors has demonstrated enhanced T-cell infiltration into tumor cores and improved efficacy in clinical studies. Similarly, drugs targeting adenosine pathways (e.g., CD73, CD39, or A2A receptors) aim to dismantle the chemical barriers established by CAFs, representing a current hotspot in immunotherapy (81–83).

Synergistic Effects: Combination with Immunotherapy Single calibration strategies often prove insufficient for managing complex diseases. The most promising approach involves combining these strategies with existing immunotherapies—particularly immune checkpoint inhibitors—to achieve synergistic effects.

**Figure 5:**
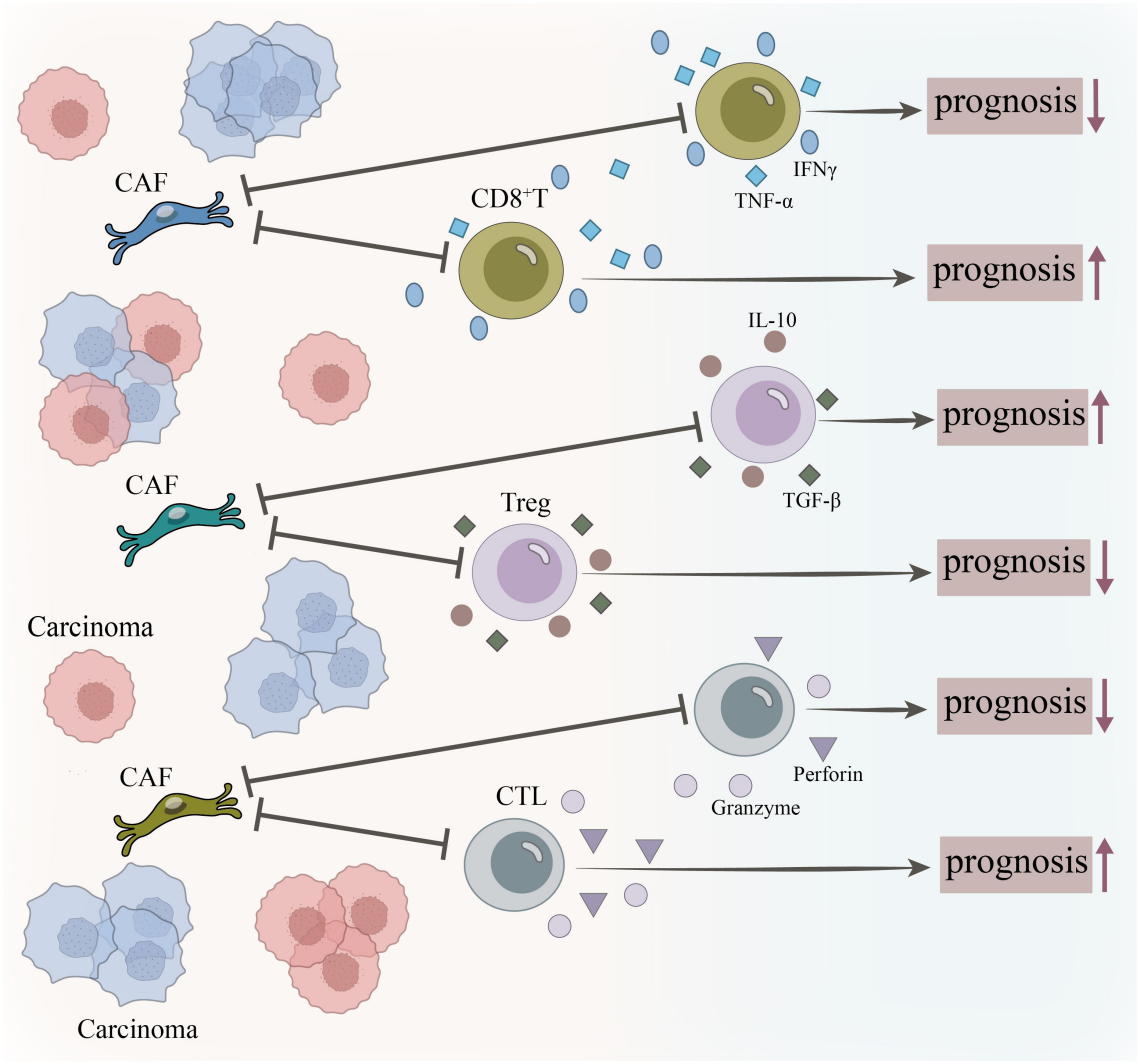
The spatial distance between CAFs and CTLs, CD8+ T cells, and Tregs aids in assessing tumor prognosis. When CAFs are distant from CD8+ T cells and CTLs, these cells cannot directly contact and kill tumor cells, and the pro-inflammatory factors secreted by CD8+ T cells struggle to act on tumor cells. Similarly, granzyme and perforin secreted by CTLs struggle to reach tumor cells. This configuration typically correlates with poorer tumor prognosis. Conversely, when these cell types are in close proximity to CAFs, tumor prognosis tends to improve. Regarding Tregs, when distant from CAFs, they are less effective at aiding tumor-associated immune cells in establishing an immune escape microenvironment, thereby favoring treatment outcomes. However, when Tregs are near CAFs, tumor prognosis is adversely affected.

Converting ‘cold’ tumors into ‘hot’ tumors: Depleting FAP+ CAFs or degrading the extracellular matrix (ECM) has been shown to increase CD8+ T cell infiltration within tumors. Building upon this, the use of PD-1/PD-L1 inhibitors can more effectively reverse the exhausted state of these T cells, thereby transforming an immune-rejecting ‘cold’ microenvironment into an immune-inflammatory ‘hot’ microenvironment (84, 85). The F5-CAF subpopulation (marked by genes COL1A2, COL4A1, COL4A2, CTGF, and FSTL1) was found to be enriched within and around tumor nests and was strongly associated with tumor stemness. Spatial analysis revealed that the microenvironment harboring F5-CAFs exhibited restricted infiltration of immune cells, including CD8+ T cells, demonstrating an “immune exclusion” signature. Although the physical distance between F5-CAFs and CD8+ T cells was not directly quantified, the study found that F5-CAFs co-localized with M2 macrophages and exhibited fewer CD8+ T cells in their enriched regions, suggesting they may hinder CD8+ T cell infiltration by shaping an immunosuppressive microenvironment. Furthermore, the F5-CAF score calculated via ssGSEA significantly correlated with poor patient prognosis, with patients exhibiting high F5-CAF scores or high F5-CAF numbers demonstrating shorter overall survival (33). CTHRC1+ CAFs also exhibit spatial exclusion from CD8+ T cells. Spatial transcriptomics analysis revealed that regions enriched with CTHRC1+ CAFs (such as the spatial microenvironment Niche_4) show minimal CD8+ T cell infiltration. These regions are simultaneously enriched with regulatory T cells and M2 macrophages, further reinforcing the immunosuppressive state. Scoring CTHRC1+ CAFs via ssGSEA revealed that high scores correlated strongly with advanced tumor stages and poor patient prognosis. Furthermore, the study validated the positive correlation between CTHRC1+ CAFs and CD8+ T cell exclusion using the TIDE database, demonstrating that this CAF subtype promotes tumor immune escape by suppressing cytotoxic T cell infiltration (86). However, due to the challenges in widespread adoption of spatial transcriptomics and its high cost, comprehensive comparisons between immune infiltration and tumor treatment efficacy remain lacking (**Figure 5**).

Precision Delivery via Nanotechnology to overcome off-target toxicity and achieve precise regulation, nanomedicine strategies have emerged. Researchers have engineered nanoparticles that specifically recognize and enrich on the surface of CAFs or in the tumor stroma to deliver siRNA for silencing pathogenic genes in CAFs (e.g., TGF-β siRNA) or to carry drugs that modulate CAF activation status for direct reprogramming of CAFs. This highly specific intracellular delivery enables precise reprogramming of the CAF phenotype, effectively dismantling the pathological barriers they construct. It offers a novel approach to restore immune cell infiltration and function while reversing resistance to immunotherapy (75, 87, 88).

## Conclusions and outlook

As the core “yardstick” of the immune microenvironment, fibroblasts actively regulate immune responses through functional heterogeneity, spatial distribution, and cellular interactions. Dysfunction in these processes drives pathological pathways such as immunosuppression, inflammation, and fibrosis. The essence of targeted therapy lies in ‘calibrating’ this dysregulated system. Future objectives focus on establishing precise, clinically applicable “metrology” systems for the immune microenvironment. Technologically, single-cell and spatial multi-omics integrate molecular and spatial information, revealing spatial relationships between fibroblasts and immune cells. For instance, the distance between FAP^+^ CAFs and CD8^+^ T cells serves as a predictive biomarker for treatment efficacy. *In vivo* imaging allows for non-invasive dynamic monitoring. Research paradigms are shifting from analyzing “cell subpopulations” to examining “functional states” and “cellular niches,” with the integration of AI to decipher regulatory networks and identify targets. Through interdisciplinary convergence, bioinformatics and AI synthesize multi-omics data to optimize therapies; biomaterials facilitate precision drug delivery; and clinical practice will incorporate fibroblast biomarkers for microenvironment stratification and personalized treatment.

Despite challenges such as tissue specificity, the establishment of a fibroblast-centric “metrology” system will advance precision medicine. This will ultimately enable dynamic interpretation and “calibration” of dysregulated microenvironments, reshaping therapeutic pathways.
